# Role of Actin-Binding Proteins in Skeletal Myogenesis

**DOI:** 10.3390/cells12212523

**Published:** 2023-10-25

**Authors:** Mai Thi Nguyen, Raju Dash, Kyuho Jeong, Wan Lee

**Affiliations:** 1 Department of Biochemistry, Dongguk University College of Medicine, 123 Dongdae-ro, Gyeongju 38066, Republic of Korea; nguyenmainhp@gmail.com (M.T.N.); khjeong@dongguk.ac.kr (K.J.); 2 Department of Anatomy, Dongguk University College of Medicine, 123 Dongdae-ro, Gyeongju 38066, Republic of Korea; rajudash.bgctub@gmail.com; 3 Department of New Biology, Daegu Gyeongbuk Institute of Science & Technology (DGIST), Daegu 42988, Republic of Korea; 4 Channelopathy Research Center, Dongguk University College of Medicine, 32 Dongguk-ro, Ilsan Dong-gu, Goyang 10326, Republic of Korea

**Keywords:** actin-binding proteins, actin dynamics, myogenesis, differentiation, proliferation, non-coding RNA

## Abstract

Maintenance of skeletal muscle quantity and quality is essential to ensure various vital functions of the body. Muscle homeostasis is regulated by multiple cytoskeletal proteins and myogenic transcriptional programs responding to endogenous and exogenous signals influencing cell structure and function. Since actin is an essential component in cytoskeleton dynamics, actin-binding proteins (ABPs) have been recognized as crucial players in skeletal muscle health and diseases. Hence, dysregulation of ABPs leads to muscle atrophy characterized by loss of mass, strength, quality, and capacity for regeneration. This comprehensive review summarizes the recent studies that have unveiled the role of ABPs in actin cytoskeletal dynamics, with a particular focus on skeletal myogenesis and diseases. This provides insight into the molecular mechanisms that regulate skeletal myogenesis via ABPs as well as research avenues to identify potential therapeutic targets. Moreover, this review explores the implications of non-coding RNAs (ncRNAs) targeting ABPs in skeletal myogenesis and disorders based on recent achievements in ncRNA research. The studies presented here will enhance our understanding of the functional significance of ABPs and mechanotransduction-derived myogenic regulatory mechanisms. Furthermore, revealing how ncRNAs regulate ABPs will allow diverse therapeutic approaches for skeletal muscle disorders to be developed.

## 1. Introduction

**Figure 1:**
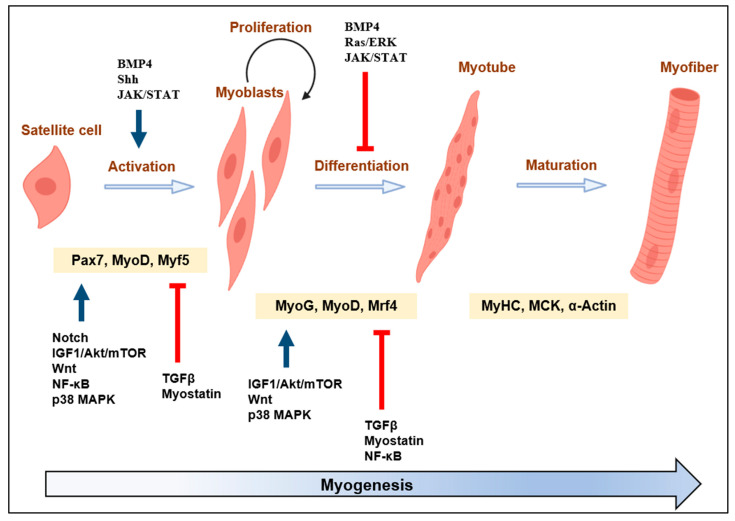
Schematic view of myogenesis. During myogenic differentiation, quiescent myogenic precursor (satellite) cells are first converted to skeletal muscle lineage myoblasts. Next, the cell cycle progression, cell proliferation, myogenic transcriptional activation, and morphological changes, such as differentiation and fusion into multinucleated myotubes, are subsequently promoted in myoblasts. Specific myogenic regulatory factors and critical signaling molecules in myogenic processes are illustrated. For the full names of each protein, see the abbreviation section.

Skeletal muscle is an abundant tissue, constituting about 40% of human body weight, and is critical for various vital functions, including locomotion, force generation, energy storage, respiration, and metabolism [1,2]. Maintaining skeletal muscle homeostasis relies on the intricate process of myogenesis, which is pivotal for the development, growth, and regeneration of skeletal muscle fibers [3]. This process is meticulously orchestrated by multistage events encompassing progenitor cell proliferation, activation of myogenic factors, differentiation, and the fusion of cells to form myotubes (Figure 1). Moreover, these sequential events are further regulated by complex networks that govern intercellular signaling and expression of myogenic regulatory factors (MRFs) [4,5]. Hence, dysregulation of skeletal myogenesis results in muscle wasting, characterized by an overall decline in muscle mass, strength, quality, and regenerative capacity [6,7]. In this aspect, MRFs and their functional modulators have been studied as promising targets for various muscular disorders. For many years, numerous classical signaling molecules and canonical signaling pathways, such as PI3K/Akt/mTOR [8], p38 MAPK [9], TGFβ/myostatin/activin [10,11], Wnt [12,13], Notch [14], BMP [12,13], Shh [12,13], JAK/STAR [15], and NF-κB [16], have been suggested as master regulators of skeletal myogenesis via modulating MRFs expression. However, in spite of these efforts, treating muscle wasting remains challenging due to its complexity and lack of understanding. Therefore, insight into skeletal myogenesis from a molecular and cellular level can contribute to developing novel therapeutic strategies against muscle atrophy by identifying specific target molecules and pathways.

Over the last decade, there has been a growing understanding of the relationship between mechanotransduction, which is the conversion of mechanical stimuli into biochemical signals, and the dynamic properties of the actin cytoskeleton during skeletal myogenesis. Actin dynamics, including actin polymerization, depolymerization, and a range of other cytoskeletal processes, not only facilitate physical changes necessary for cellular structural reconfiguration but also initiate mechanotransduction, which activates myogenic genes and differentiates progenitor cells [17,18]. This complicated connection significantly impacts myogenic transcription programs, cytoskeletal organization, cell proliferation, and differentiation [19,20]. Consequently, dynamic reconfiguration of actin filaments alters cell morphology, allowing myoblasts to change flattened shapes and subsequent merging of cell membranes, which facilitates myoblast fusion [20,21,22]. As a key point to emphasize, a large number of actin-binding proteins (ABPs) control actin dynamics, including polymerization, monomer sequestration, depolymerization, nucleation, capping, budding, cross-linking, and stabilization [23]. From this perspective, ABPs are now recognized as critical players in skeletal myogenesis via mechanotransduction, which triggers proliferation, migration, differentiation, and morphogenesis in cells [20,21,22]. This review aims to comprehensively summarize the functions of ABPs in skeletal muscles, emphasizing recent advancements concerning myogenesis and their implications in muscular disorders. In addition, ABPs in the myogenic process are also known to be modulated by many epigenetic modifiers, particularly non-coding RNAs (ncRNAs), including long non-coding RNAs (lncRNAs) and microRNAs (miRNAs). In this context, we also provide evidence that ncRNAs have an influence on the expression of ABP genes, thereby affecting actin cytoskeleton dynamics and myogenesis.

## 2. Overview of Actin Cytoskeleton Dynamics, ABPs, and Myogenesis

**Figure 2:**
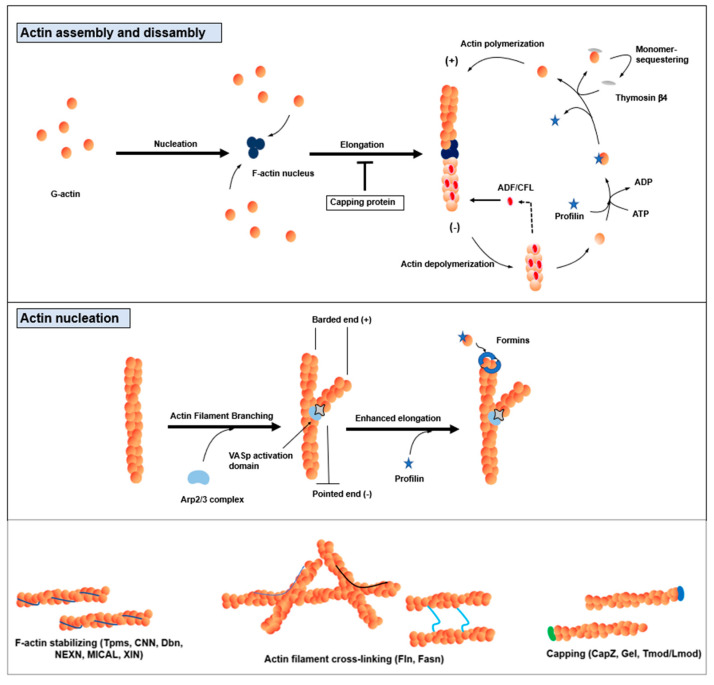
Roles of various ABPs on the regulation of actin cytoskeleton dynamics. ABPs are divided into seven groups based on their cellular function as described in Section 3; (1) actin polymerization (PFN); (2) monomer-sequestering (Thymosin β4); (3) actin depolymerization (Des, CFL, TWF, WDR1, CAP); (4) nucleation sites in actin branching (Arp2/3, WASP, N-WASP, Formins, Abp1, Pan1, CTTN); (5) capping (CapZ, Gel, Tmod/Lmod); (6) actin filament cross-linking (Fln, Fasn); and (7) F-actin stabilizing (Tmps, CNN, Dbn, NEXN, MICAL, XIN). For the full names of each protein, see the abbreviation section.

The actin cytoskeleton is characterized by its remarkable capacity for continuous restructuring through the processes of actin polymerization and depolymerization. Actin polymerization involves the addition of ATP-actin monomers (globular actin or G-actin) at the rapidly growing barbed end (+end). In contrast, ADP-actin monomers are consistently released from the slowly growing pointed end (-end) of the elongated actin polymers (filamentous actin or F-actin) [23,24,25]. This dynamic assembly and disassembly of actin filaments are strictly regulated both spatially and temporally by ABPs, which are crucial components for maintaining the balance between cellular G-actin and F-actin [23,24,25]. Thus, the intricate interplay between actin dynamics and ABPs ensures the precise control of cellular architecture and function. Approximately a quarter of all cellular proteins are attributed to ABPs, and these proteins can be classified into conserved families based on their specific cellular functions on actin dynamics (as depicted in Table 1 and Figure 2). 

The significance of actin dynamics extends beyond mere structural alterations; it encompasses essential processes during skeletal myogenesis. Notably, actin dynamics facilitate myoblast recognition and fusion during cell-cell interactions [20,21,22]. This phenomenon is crucial for the formation of multinucleated myotubes and the precise alignment of myotubes with their respective attachment sites. Accumulating evidence has also underscored the critical role played by ABPs in regulating ADP-actin/ATP-actin exchange during skeletal muscle development and regeneration [26,27,28,29,30,31]. In both in vivo and in vitro models, researchers have applied inhibitory agents targeting actin and ABPs, such as latrunculin or cytochalasin, to assess the impact of actin dynamics on myogenesis [22,32,33]. These interventions have revealed that myoblasts treated with monomeric actin-binding drugs, such as cytochalasin or latrunculin, undergo actin depolymerization, thereby impeding myoblast fusion by disrupting F-actin formation at the myoblast fusion site [33,34,35]. These findings demonstrate the indispensable role of actin dynamics in myogenesis. Moreover, pharmacological agents like jasplakinolide, which bind to F-actin and promote actin polymerization, have further elucidated the essential role of actin remodeling in the regulation of myogenesis [36]. Treatment with jasplakinolide has been shown to dramatically suppress actin dynamics in premyofibrils and mature myofibrils, providing additional evidence of the significance of actin dynamics in skeletal muscle homeostasis [36]. Consequently, various medications targeting ABPs have been developed with the aim of modulating cellular motility, muscle contraction, and myogenesis, potentially offering therapeutic avenues for muscle disorders [22,32]. 

**Table 1:** Roles of major ABPs in actin cytoskeleton dynamics.

Protein Group	Actin-Binding Protein	Main Function	Refs.
Proteins regulating F-actin assembly	Profilin (PFN)	Binds to and sequesters actin monomer and promotes actin polymerization.	[37,38]
Proteins for F-actin dissambly	ADF/cofilin family (Destrin (Des), Cofilin (CFL))	Bind both F-actin and G-actin, leading to depolymerization from the pointed ends	[39,40]
	WD-repeat domain 1 (WDR1)	Major cofactor of ADF/CFL	[41,42]
	Twinfilin (TWF)	Binds and sequesters ADP-actin monomer, blocks filament elongation, and caps activity	[43,44,45]
	Cyclase-associated protein (CAP)	Required to activate adenylyl cyclase, which binds to G and F actin, ADF/CFL partner, regulating actin filament dynamics	[46,47]
Monomer-sequestering proteins	Thymosinb4 (Tb4)	Sequesters actin monomer, and prevent it from engaging in the polymerization reactions	[25]
	Myocardin-Related Transcription Factor (MRTF-A)	Controls G-actin/F-actin balance, generating a closed link between actin dynamics and gene expression.	[48]
Proteins for actin nucleation	Formins	Enables the elongation of actin filament at the barded end	[49]
Proteins for nucleation sites in actin branching	Actin-related protein-2/3 (Arp2/3)	Promotes actin nucleation to generate a new branched (daughter) actin network by binding to the side of the existing filament (mother)	[50,51]
	Wiskott—Aldrich syndrome protein (WASP), suppressor of cyclic AMP repressor (SCAR or WAVE)	Activate Arp2/3 complex to induce the branching of a new filament	[52,53]
	*S. cerevisiae* actin-binding protein-1 (Abp1), Pan1, and Cortactin (CTTN)	Promote and stabilize Arp2/3-mediated branches	[50,54]
Capping proteins	CapZ	Binds barbed ends to stop filament growth	[55,56]
	Gelsolin (Gel)	Promotes the nucleation step of actin polymerization	[57]
	Tropomodulin (Tmod)/Leiomodin (Lmod)	Cap the pointed ends of the actin-based thin filaments in striated muscle	[58]
Actin filament cross-linking proteins	Fascin (FSCN)	Promotes filopodia assembly, and stable actin bundles	[59]
	Filamin (Fln)	Binds F-actin into orthogonal branches, cross-link F-actin	[60]
F-actin stabilizing proteins	Calponin (CNN)	Inhibit actomyosin ATPase, regulate and stabilize actin filament motility	[61]
	Drebrin (Dbn)	Binds to the F-actin side and promotes F-actin formation	[62]
	Microtubule associated monooxygenase (MICAL)	Induces redox reactions on F-actin, makes certain disaggregation, and prevents polymerization	[63,64]
	Nexilin (Nelin, NEXN)	F-actin cross-linking activity through binding along the sides of F-actin	[65]
	XIN-repeating protein (XIN)	Prevents depolymerization by binding to F-actin	[66]
ABPs related to muscle contraction	α-actinin, myosin IIs (NM IIs), Nebulin, Tropomyosins (Tpms)	Stabilize and regulate F-actin	[67,68,69,70,71,72]

Although the significance of actin cytoskeleton dynamics and its regulatory networks have been extensively studied, the specific roles played by each ABP in skeletal myogenesis are still unclear. In the following section, we discuss recent advances in the understanding of how ABPs contribute to skeletal myogenesis and suggest future research directions. Through this process, we aim to shed light on the potential applications of ABPs in skeletal muscle disorders.

## 3. Roles of ABPs on Actin Remodeling and Skeletal Myogenesis 

### 3.1. Proteins Regulating F-Actin Assembly 

In the formation of individual muscle fibers, a founder cell serves as the starting point, harboring the developmental instructions that dictate the distinct characteristics of each muscle. Many studies have highlighted the crucial role of actin polymerization in the fusion and differentiation of myoblasts in skeletal muscle [17,18]. This section explores the pivotal role played by profilin, an ABP, in regulating actin assembly and its impact on the intricate events underlying muscle development and differentiation.

#### Profilin (PFN)

PFN serves as a regulator of de novo actin assembly in actin polymerization by binding to and sequestering actin monomers to the filament-barbed end [37]. The PFN gene family includes two isoforms, profilin I (PFN1) and profilin II (PFN2) [73]. In vertebrates, profilin I (PFN1) is expressed ubiquitously, whereas profilin II (PFN2) is highly expressed only in skeletal muscles, brain, and kidney [74,75]. PFN1 and PFN2 are found early in development and gradually decrease during the differentiation of myoblasts and satellite cells [76,77,78]. Gopinath et al. reported that PFN1 inhibited myogenic differentiation by binding to RhoA/Rac1 and repressing RhoA/Rac1 at the transcriptional level [79]. RhoA and Rac1 have been shown to positively regulate myogenic differentiation through serum response factor (SRF) and β-catenin/TGF, which activate the expression of MyoD, thereby promoting myogenesis [79,80]. Moreover, the interaction of PFN1 with Cdc42, a GTPase protein, has been shown to stimulate PAK/JNK signaling pathways, altering cytoskeletal dynamics and inhibiting myogenic differentiation [81]. In a study by Kooij et al., overexpression of PFN2a in *Drosophila* resulted in reduced climbing ability of flight muscles, diminished flight capability, and elongated thin filaments [82]. In addition, PFN2a was found to inhibit cell proliferation, induce apoptosis, repress myogenic differentiation in C2C12 myoblasts, and disrupt sarcomere structure assembly [83]. Mechanistically, PFN2a reduced nuclear localization of histone deacetylase 1 (HDAC1) and induced p53 expression. This effect led to the activation of the tumor suppressor gene p21 and the inhibition of cyclin–CDK phosphorylation of the Rb complex, resulting in growth arrest, apoptosis, and reduced myogenic differentiation [83]. These findings underscore the significance of PFN-dependent actin polymerization and its regulatory role in muscle development, particularly in myogenic differentiation. The multifaceted impact of PFN on myogenesis, apoptosis, and sarcomere assembly makes it a promising candidate for targeted therapeutic interventions in skeletal muscle disorders.

### 3.2. Proteins Regulating F-Actin Disassembly

#### 3.2.1. Actin-Depolymerizing Factor/Cofilin Family Proteins (ADF/Cofilin)

The intricate dynamics of actin in muscle development are closely regulated by proteins that control the availability of G-actin for its incorporation into F-actin. A pivotal group of proteins within this context is the ADF/CFL family, which significantly influences myoblast fusion and regeneration by interacting with F-actin and modulating its transition to G-actin [84]. The ADF/CFL family, including nonmuscle-type CFL1, muscle-type CFL2, and Destrin (Des), is ubiquitous across eukaryotes, with heightened expression levels observed in mammals [85]. During muscle development in healthy mice, a noteworthy transition occurs from nonmuscle-type CFL1 to muscle-type CFL2. CFL1 is predominantly present during the early stages and diminishes as muscle development progresses, while CFL2 exhibits increased expression levels during muscle differentiation and fusion [86,87,88,89,90]. This dynamic expression shift implies that CFL1 plays a role in the initial phases of muscle growth, whereas CFL2 exerts a more pronounced influence on muscle development and maintenance. The indispensable nature of CFL1 and CFL2 is evident from the fact that mice lacking CFL1 succumb during embryonic development, while those lacking CFL2 succumb at eight weeks of age [26,90]. 

Experimental evidence from Drosophila underscores the significance of CFL in muscle development. Loss of CFL in *Drosophila* muscles leads to impaired actin remodeling, aggregation of sarcomeric proteins, and aberrations in sarcomere addition during skeletal muscle development [91]. Recent research by Sun et al. has provided further insights, demonstrating that reduced CFL1 levels during myoblast differentiation notably enhance the expression of essential myogenic regulatory genes, such as MyoD, MyoG, and MyHC, as markers of myogenic differentiation. Conversely, CFL1 overexpression suppresses the differentiation of bovine primary myoblasts [26]. Notably, mutations in the CFL2 gene have been associated with nemaline myopathy, characterized by delayed motor development and atrophied muscle fibers [91,92,93]. Studies in CFL2-knockout mice have unveiled abnormal F-actin accumulation and progressive disruption of the sarcomeric architecture in skeletal muscles [93,94,95,96]. Additionally, CFL2 knockdown in C2C12 myoblast cells has been shown to inhibit myogenic regulatory factors (MyoG and MyoD), MyHC isoforms (I, IIa, IIx, and IIb), and myotube formation [26,27]. Suppression of CFL2 expression leads to F-actin accumulation and nuclear translocation of YAP in the Hippo pathway, promoting cell proliferation and inhibiting myogenic differentiation in C2C12 cells [90]. In accordance with previous findings, silencing CFL2 expression results in the downregulation of p38 MAPK, CBP, AMPKα1, and MEF2C pathways while upregulating ERK2 expression. This cascade of molecular events culminates in reduced myogenic differentiation in C2C12 cells [27]. The implications of CFL2 dysregulation extend to its correction, presenting an essential avenue for investigation in the context of muscle atrophy. Moreover, restoring CFL2 dysregulation in muscle-related disorders accentuates the potential for therapeutic interventions targeting these proteins.

#### 3.2.2. WD-Repeat Protein 1 (WDR1)

In addition to the ADF/CFL family’s crucial involvement in actin depolymerization, an increasing number of ABPs serve as essential cofactors in this intricate process. WD-repeat protein 1 (WDR1), an actin-binding protein, is identified as a primary cofactor of ADF/CFL-decorated actin filaments and promotes the disassembly and capping of the barbed ends of the severed actin filaments [41]. In human skeletal muscle, WDR1 protein was found to be highly expressed during exercise after muscle disuse, indicating that WDR1 is associated with adaptive sarcomere reorganization [97]. While WDR1 has been studied in limited fields, its specific role in skeletal myogenesis and myopathies remains unknown.

#### 3.2.3. Cyclase-Associated Actin Cytoskeleton Regulatory Protein (CAP)

Another noteworthy cofactor within the ADF/CFL family is CAP proteins, which can interact with the ADF/CFL family proteins spatiotemporally and control F-actin depolymerization and CFL release from G-actin for further actin dynamics [28,98]. One of CAP isoforms, CAP1, is highly expressed in nonmuscle cells during the early stages of myogenic differentiation but exhibits reduced expression as differentiation progresses [99,100]. Conversely, CAP2 is exclusively expressed in the brain and skeletal muscles, maintaining high expression levels throughout differentiation and regeneration [99,100]. CAP1 deficiency results in an altered cellular morphology, increased cell size and nuclei, and aberrant F-actin organization, leading to the inhibition of myogenic differentiation [100]. Likewise, mutations in CAP2 induce structural changes in skeletal muscle, characterized by reduced muscle strength, delayed maturation of motor functions, and the formation of ring fibers due to elusive disruptions of F-actin depolymerization [28]. Importantly, CAP2 collaborates with CFL2 in regulating the transition of α-actin isoforms to skeletal muscle (α-SKA) from cardiac (α-CAA) and smooth muscles (α-SMA), a crucial process in myoblast differentiation and fusion [93]. In this regard, knockout of CAP2 in mice promoted abnormal F-actin formation because of α-SMA and α-CAA accumulation, thereby leading to ring fiber formation associated with impaired motor function and histopathological alterations [28]. In addition, CAP2 expression is downregulated in obesity-induced muscle atrophy, emphasizing its significance in muscle homeostasis [101]. Therefore, the coordinated expression patterns of CAP1, CAP2, and their partner CFL2 during differentiation contribute to a comprehensive understanding of skeletal muscle regeneration and myoblast fusion processes.

#### 3.2.4. Twinfilin (TWF)

TWF is an actin-depolymerization protein with sequence homology to the ADF/CFL family [43,101]. Two biochemically distinct TWF isoforms, TWF1 and TWF2, are usually found in mammals, where TWF1 is ubiquitously expressed in nonmuscle tissues, whereas TWF2 is highly expressed in cardiac and skeletal muscle [102,103]. During myogenic differentiation, TWF2 expression remains stable throughout the differentiation period [31]; however, TWF1 expression increases in the early stage of myoblast fusion (the highest expression on differentiation day three and then gradually decreases) [31]. Nguyen et al. recently demonstrated that knockdown of TWF1 induced F-actin accumulation, leading to increased nuclear YAP and consequent impediments in myogenic differentiation, primarily through enhanced cell proliferation in C2C12 myoblasts [31]. However, further characterization of the role of TWF1 in animal models and patients with skeletal muscle diseases is essential to deepen our understanding. 

### 3.3. Monomer-Sequestering Proteins

#### 3.3.1. Thymosin β4 (Tβ4)

Tβ4 is a small N-terminally acetylated polypeptide (4.9 kDa), which functions as a significant intracellular G-actin-sequestering peptide and inhibits the assembly of actin fibers in cells [104]. While initially recognized for its intracellular function, Tβ4 has recently gained recognition for its extracellular roles in cardiovascular regeneration, wound healing, and angiogenesis [105,106,107]. Several studies found that Tβ4 was upregulated in the skeletal muscles of dystrophin-deficient mdx mice [108,109] and a mdx-derived myoblastic cell line [110]. Moreover, Tβ4 mRNA levels surge during myotube differentiation of C2C12 cells and significantly increase in the early stages of muscle fiber regeneration and inflammation within injured skeletal muscles [111,112]. These findings suggest that elevated Tβ4 levels facilitate myoblast migration to regenerating muscle regions. However, further research is needed to uncover potential Tβ4 receptors and elucidate the mechanisms through which intracellular Tβ4 exerts its cellular functions.

#### 3.3.2. Myocardin-Related Transcription Factor (MRTF)

The MRTF family is another G-actin-sequestering factor that plays a pivotal role in modulating various biological processes by influencing actin cytoskeleton rearrangement [48]. MRTFs, along with other actin-binding proteins like thymosin β4 (Tβ4), profilin, DNAse I, and capping proteins, sequester unbound G-actin, effectively preventing spontaneous actin polymerization [24,113]. MRTFs are initially located in the cytoplasm, where they bind specifically to G-actin, interfering with their nuclear translocation via interaction with the nuclear transport receptor importin α/β. This interference inhibits the nuclear translocation of MRTFs. When external stimuli induce actin polymerization, the cytoplasmic pool of G-actin decreases, prompting the translocation of G-actin-free MRTFs into the nucleus, where they become transcriptionally active [114,115,116]. 

MRTF-A is implicated in skeletal muscle differentiation and homeostasis and is required for myogenesis in vivo and in vitro [117,118,119]. It has been evidenced by studies involving its overexpression or knockout, which consistently reveal impairments in the differentiation process [117,118,119]. Moreover, mice lacking MRTF-A display viability but exhibit irreversible hypoplasia in their skeletal muscles [120]. The correlation between augmented MRTF-A protein levels and the regenerative response to skeletal muscle injuries within satellite cells (SCs) is a noteworthy observation [121,122]. One of the critical mechanisms by which MRTFs contribute to actin dynamics is through their interaction with the serum response factor (SRF), partnering with it to regulate the transcription of genes associated with actin cytoskeleton organization and function via binding to a conserved CArG box sequence (CC(A/T)6GG) in the promoter region [117,123]. Silencing MRTF-A in C2C12 myoblasts hampers the expression of genes linked to SRF-targeted differentiation, underscoring its significance in the process [118]. The MRTF-SRF transcription factor combination triggers numerous targets, encompassing many genes specific to muscle function [124,125]. Interestingly, MRTF-A and RhoA, an activator of MRTF-A, exhibit similar patterns of expression and functional behavior; sustained RhoA overexpression induces complications in differentiation, mirroring the effects of increased MRTF-A levels [117,118]. Furthermore, the downregulation of MRTF-A expression during myoblast fusion, coupled with active RhoA, results in differentiation defects in vitro [119,126]. The interplay between MRTF-A, RhoA, and their impact on muscle injury and muscular dystrophy has been a subject of prior research [117,118]. 

Previous investigations into molecular signaling pathways have demonstrated that MRTF-A and PAX7, a transcription factor associated with satellite cells (SCs), are increased in SCs during muscle regeneration [127]. SCs-specific ablation of MEF2 activating motif and SAP domain-containing transcriptional regulator (MASTR), another member of the MRTF family, disrupts the myogenic program primarily by affecting the transcriptional activity of MyoD. This disruption becomes particularly pronounced in MASTR and MRTF-A double knockout mice, wherein aberrant differentiation significantly increases MyoD expression [121]. RNA-seq studies have further established a strong correlation between MRTF-A levels and myogenic genes, with decreasing MRTF-A in C2C12 cells resulting in reduced PAX7 expression. This correlation holds during myoblast differentiation and injury-induced muscle repair, suggesting the role of MRTF-A as a regulator of PAX7 during myoblast commitment to differentiation [127]. Notably, manipulating MRTF-A levels leads to distinct outcomes: increased MRTF-A promotes myoblast proliferation while inhibiting differentiation and the expression of MyoD and MyoG [127]. Overall, MRTF-A emerges as a novel regulator of PAX7 during myoblast commitment to differentiation, potentially offering avenues for manipulating muscle stem cell fate and exploring stem cell-based therapies for muscle degenerative diseases.

### 3.4. Proteins for Nucleation Sites in Actin Branching

#### 3.4.1. Actin-Related Protein-2/3 (Arp2/3) and WASP/WAVE 

During *Drosophila* myogenesis, the multinucleated myotube elongates in two directions along a linear axis towards its specific attachment sites in the epidermis, called tendon cells. At the ends of the myotube, extensive filopodia, similar to neuronal growth cones, search the environment for their attachment sites. Migrating myotubes have been shown to respond to signals from the tendon cells, which are specified by the Stripe transcription factor, and induce attraction and adhesion of the approaching myotube. Since genetic studies demonstrated a role for Arp2/3-mediated actin polymerization in myoblast fusion, significant efforts have been directed at understanding the precise function of actin cytoskeletal rearrangement in the fusion process [128]. 

The Arp2/3 complex, a core participant in actin polymerization, is required to construct new filament networks by promoting actin nucleation for the formation of a new branch (daughter) of the actin network from an existing filament (mother) in cells [51,129]. This process is pivotal in myoblast fusion, an indispensable facet of muscle development [51,129]. Branch formation is initiated by the Arp2/3 complex when activated by nucleation-promoting factors (NPFs), which are classified as type I and II [50]. Type I NPFs encompass the Wiskott-Aldrich syndrome protein (WASP) and the WASP family verprolin-homologous (WAVE) proteins [52,53]. On the other hand, type II NPFs include metazoan cortactin (CTTN), *S. cerevisiae* actin-binding protein-1 (Abp1), and Pan1 [51,53,129]. In actin assembly, activation of Arp2/3 by the Cdc42/Rac signaling pathway is directly modulated by WASP/WAVE [130,131], and actin nucleation processes are also regulated by the interaction of WASP/WAVE with Grb2, Toca, Nck, IRSp53, and WASP-interacting protein (WIP) [132,133,134]. In contrast, WAVE always works in four other complex components, including Abi, Nap1/Hem2/Kette, HSPC300/Brick1, and Sra1/Cyfip1, named the WAVE regulatory complex (WRC) [135]. 

The essential involvement of Arp2/3 and the WASP/WAVE family in myogenesis has been previously documented in *Drosophila* [129] and mice [136,137,138]. N-WASP is expressed in the early phase of embryonic development in mice and implicated in filopodia formation required for cell movement and actin-based motility [139,140]. Inactivation of N-WASP in mice results in early embryonic lethality because of developmental delay [141]. Furthermore, interference of N-WASP function both in vivo and in vitro showed that myoblasts failed to fuse and form multinucleated myotubes [136,137,138]. In Cdc42/Rac mutated embryonic mice, the F-actin and Arp2/3 complex expressions were significantly lower than in the control mice, as well as other characteristics, such as thinner muscle fibers and myoblast fusion [142]. Therefore, the role of N-WASP in the regulation of actin dynamics is proposed to be one of the key mechanisms intervening in myotube formation and fusion. 

Positive regulation of Kette/Nap1, a member of the WAVE actin-remodeling complex, is also necessary for correct localization and stabilization of SCAR/WAVE, allowing myoblast fusion in *Drosophila* [129]. Inhibition of Nap1 through shRNA-mediated knockdown substantially reduces the levels of two WAVE family members, WAVE1 and WAVE2. Interestingly, this inhibition results in the aberrant formation of F-actin at the plasma membrane, leading to the repression of myoblast fusion, all while leaving Abi1 levels unaffected [29]. Importantly, the transient knockdown of WAVE2 leads to inhibition of myoblast fusion, whereas WAVE1 does not play a vital role in the fusion of C2C12 myoblasts [29]. Furthermore, it is interesting that hepatocyte growth factor (HGF), a well-documented activator of proliferation and migration during skeletal muscle regeneration, elicits non-directional and directional cell migration and induces the condensation of N-WASP and WAVE2 in the lamellipodia of C2C12 myoblasts, further underscoring their importance in muscle development [30]. Collectively, the Arp2/3 complex and WASP/WAVE proteins play a complementary role in the orchestration of actin nucleation and branching, critical processes in muscle development. Moreover, N-WASP emerges as a key player in myotube formation during muscle development. Research into this field promises to provide valuable insights into muscle biology and potential therapeutic applications.

Grb2 is an N-WASP activator that promotes actin polymerization by binding to the proline-rich domain of N-WASP [143]. However, several studies have reported that Grb2 acts as a negative modulator of myogenic differentiation, which is opposite to the function of N-WASP [137,144,145]. Mitra et al. suggested that the binding of Grb2 to N-WASP caused mislocalization or inhibition of N-WASP-induced defects in actin polymerization during myogenesis [137]. In addition, the downregulation of EGFR, an upstream effector of Grb2 [146], could facilitate myoblast differentiation by inhibiting Grb2 function [147]. These findings indicate that the effect of Grb2/N-WASP on muscle differentiation might be associated with the EGFR signaling cascade. On the other hand, the reduction of Toca-1 in C2C12 cells, a transducer of Cdc42-mediated actin polymerization, led to a substantial decrease in myogenic differentiation due to a deficiency in N-WASP function [148]. 

#### 3.4.2. Formin Homology Domains (FHOD)

Formins represent a diverse group of proteins that play pivotal roles in actin polymerization through their characteristic formin homology domains (FH1 and FH2) [149]. Their essential function is elongating preexisting actin filaments by binding to the barbed ends, protecting them from capping proteins like CapZ. Also, formins interact with profilin-actin complexes to facilitate the addition of new actin subunits to growing filaments [150]. In eukaryotic species, multiple formin proteins are expressed during postnatal heart development and in the *Drosophila* flight muscle [151,152]. Among these formins, members of the FHOD family have garnered significant attention. FHOD3, for instance, exhibits a broad expression profile in skeletal muscle and predominantly accumulates within sarcomeric structures. Conversely, FHOD1 is found in muscle and non-muscle cells, particularly in cardiomyocytes [153,154]. Notably, when examining the flight muscle myofibrils in *Drosophila*, the targeted silencing of *Fhos*, a gene encoding FHOD proteins, results in significant disruptions [155]. Although several lines of evidence reported that FHOD3 plays a fundamental role in sarcomere organization in mice [155,156,157,158], intriguingly, mice lacking FHOD3 showed no significant differences between knockdown and control neonates [156]. These findings underscore the complexity of formin involvement in muscle development and suggest that other factors may compensate for the absence of FHOD3.

#### 3.4.3. Disheveled-Associated Activator of Morphogenesis (DAAM)

Another prominent member of the sarcomeric formin family is DAAM, which plays a crucial role in heart development, further highlighting the significance of formins in muscle biology. Its biochemical activities are akin to those of FHOD, involving the nucleation and elongation of actin filaments [159,160]. In cultured cardiomyocytes and mature cardiac myofibrils, DAAM1 localizes to Z-bands, underscoring its role in sarcomere organization in the heart [151,161]. However, its accumulation pattern in skeletal muscles differs, implying distinct functions in different muscle types [151,161]. In *Drosophila*, DAAM exhibits intricate localization patterns in flight and larval body wall muscles [154]. These findings suggest that DAAM directly participates in the early steps of myofibrillogenesis by polymerizing sarcomeric actin filaments. 

#### 3.4.4. Protein Diaphanous (Dia)

Recently, Dia, a third formin, has also been indicated to play an essential role in myofibrillogenesis in flight muscle development of *Drosophila* by regulating the length and width of each sarcomere [154,162]. Besides, Dia also plays a pivotal role in myoblast foci formation through its direct and indirect interactions with the Arp2/3 complex [163]. Similar to Dia, the *Caenorhabditis elegans* gene, called CK1-1, regulates lattice growth and maintenance in the striated muscles of *C. elegans*. However, the function of formins in muscle myogenesis is still unclear [164].

#### 3.4.5. Cortactin (CTTN)

CTTN is another actin-binding protein necessary to assemble branched actin and maintains its stability via binding with the Arp2/3 complex [165] and WASP family proteins [166,167]. In skeletal muscle, the role of CTTN extends to insulin signaling and glucose metabolism. Actin stress fibers are known to play a fundamental role in GLUT4myc translocation in response to insulin stimulation [168,169,170,171]. Intriguingly, experiments involving CTTN have demonstrated its impact on this process. Overexpression of CTTN has resulted in the accumulation of F-actin and the induction of stress fibers in L6 myotubes and C2C12 cells. These changes, in turn, facilitate GLUT4myc translocation, highlighting the pivotal role of CTTN in insulin-induced glucose uptake [168,169]. In contrast, studies involving CTTN mutations have provided further insights. Inhibition of actin stress fiber formation and the subsequent failure of GLUT4 translocation have been observed in these instances. These findings emphasize the critical role of CTTN in mediating insulin-stimulated glucose transport, with implications for glucose homeostasis and muscle function [168,169]. Beyond its role in actin dynamics and insulin signaling, the biological significance of CTTN extends to membrane repair processes and cell migration. SIRT1, a member of the sirtuin family, has been reported to participate in these cellular events [172,173]. Iwahara et al. have suggested that SIRT1 promotes membrane resealing at injury sites by deacetylating CTTN, subsequently inducing lamellipodia formation in C2C12 myoblasts [169]. This intriguing interplay between CTTN and SIRT1 underscores the complexity of CTTN’s functions in muscle biology. Even though CTTN has been mainly demonstrated to play a role in lamellipodia formation, cell migration, and endocytosis, its biological significance during skeletal myogenesis is expected to be revealed soon.

### 3.5. Capping Proteins 

During the intricate processes of actin dynamics, ATP monomers within the polymerized filament undergo hydrolysis to ADP monomers, leading to their dissociation [24]. This process necessitates the action of capping proteins, such as Gelsolin (Gel), CapZ, and Tropomodulin (Tmod)/Leiomodin (Lmods), to regulate filament length and assembly by binding to the ends of the actin filaments. Therefore, capping proteins are pivotal in cellular structure and various functions [57]. The expression and regulation of these proteins are integral in muscle development, particularly in myoblast differentiation and myofibrillar assembly.

#### 3.5.1. Gelsolin (Gel)

Gel is a Ca^2+^- and phosphoinositide-dependent severing and capping protein that regulates filament lengths by binding to F-actin in the barbed end and is predominantly expressed in skeletal and cardiac muscles [174]. Gel-regulated lateral transverse muscle size inhibits separation by limiting myoblast fusion in *Drosophila* [175]. The expression levels of Gel were found to be steadily induced during myoblast differentiation, highlighting its significant role in myofibrillar assembly [176]. This suggests that Gel has a functional and structural relevance in forming and maintaining muscle fibers, potentially impacting overall muscle physiology and function. 

#### 3.5.2. F-Actin-Capping Protein Subunit Beta 1 (CapZβ1)

Several lines of evidence implicate mutations in CapZ-β cause a lethal phenotype during the early larval stage in *Drosophila* [177]. Disruption in the physiological functions of CapZβ1 in mice, particularly through the overexpression of CapZ-β2, has been observed to result in severe myofibril disruption, ultimately leading to death [177]. In addition, a report showed that CapZ was found to be upregulated in myotubes [35]. However, despite these observations, the precise function and impact of CapZ during myogenesis remain unclear. 

#### 3.5.3. Tropomodulins (Tmods)

Tmods are members of the capping protein family, with a specialized function in capping the pointed end of actin filaments [67,68]. In mammalian skeletal muscles, the regulation of thin filament length becomes intricate due to the presence of various Tmod isoforms. Tmod1, for instance, is predominantly expressed in skeletal muscle fibers. In contrast, Tmod4 is majorly expressed in fast muscle fibers [178,179,180]. Interestingly, during muscle differentiation, there is a noticeable shift where the expression of Tmod4 can potentially replace Tmod1 [179]. Given its prominent expression in skeletal muscles, Tmod4 has been identified as a candidate gene associated with limb girdle muscular dystrophy disease [181]. During the differentiation of C2C12 cells, evidence indicates that MEF2A, a myogenic transcription factor, is significantly upregulated and binds to the promoter region of Tmod4, regulating Tmod4 expression [182]. This modulation in expression levels of Tmod isoforms plays a pivotal role in muscle development and myofibrillar assembly. Surprisingly, overexpression of Tmod1 in muscles leads to irreversible shortening of thin filaments and subsequent degeneration of myofibrils [183,184,185]. Furthermore, elevated levels of Tmod1 in fibroblasts and myoblasts significantly inhibit myogenic differentiation by reducing the expression of muscle-specific genes [186]. These observations suggest that Tmods may play a repressive role in the differentiation of myogenic cells. The potential repressive role of Tmods in myogenic differentiation highlights the need for comprehensive studies to understand their precise functions and implications in muscle disorders. 

#### 3.5.4. Leiomodin (Lmod)

Lmod, another member of the Tmod protein family, is distinguished by its three isoforms: Lmod1, Lmod2, and Lmod3 [68]. These isoforms exhibit tissue-specific expression patterns, with Lmod1 and Lmod2 being highly enriched in smooth and cardiac muscles respectively, and Lmod3 specifically expressed in skeletal muscles [68]. Similar to Tmod, Lmod proteins bind to the pointed ends of actin filaments, facilitating actin polymerization and stabilizing binucleated or trinucleated actin through three actin-binding domains [187]. Lmod3 is localized in sarcomere thin filaments and is critical in determining their length [188]. Recent evidence has demonstrated that the loss of Lmod3 function leads to lethal nemaline myopathy and severe disruption of skeletal muscle sarcomeric structure and function [189]. During differentiation, Lmod3 is induced and fosters myoblast differentiation and proliferation. Conversely, the knockdown of Lmod3 inhibits myogenesis and induces apoptosis [190]. Recent studies elucidate that MRTF/SRF and MEF2 finely regulate the expression of Lmod3 during skeletal muscle development. These form a feed-forward circuit to synchronize Lmod3 expression with sarcomeric assembly [191]. By promoting actin polymerization, Lmod3 modulates the level of G-actin in the cytoplasm, thereby releasing MRTF and augmenting MRTF and SRF activity in muscle cells [191]. It has been observed that MRTF stimulates PI3K/Akt signaling in muscle cells [192]. Consequently, SRF triggers the expression of Lmod3, perpetuating the release of more MRTF and stimulation of SRF activity. This complex interaction between PI3K/Akt and Ras/ERK pathways induces myoblast differentiation and proliferation. However, the findings necessitate further validation and research to explore the nuanced interactions and implications of Lmod in skeletal myogenesis.

### 3.6. Actin Filament Cross-Linking Proteins

#### 3.6.1. Filamins (Flns)

Flns are representative actin-cross-linking proteins, comprising Flna, Flnb, and Flnc, that play a crucial role in organizing actin filaments into filament bundles and three-dimensional networks [60,193]. Flna and Flnb are ubiquitously expressed, with their expression noted to decrease during cell differentiation. In contrast, Flnc is primarily localized in cardiac and skeletal muscles and is associated with muscle-related genes implicated in various types of muscular dystrophy [194,195]. The specificity in the distribution of Flnc underscores its significance in muscle physiology and pathology. Studies by Van der Flier et al. have demonstrated that overexpression of Flnc promotes myotube formation and myogenic differentiation. However, it also results in the formation of thinner myotubes due to alterations in the morphology of C2C12 cells [196]. The defects in Flnc have been observed to cause death shortly after birth in mice, characterized by the presence of primary myotubes and fewer muscle fibers [197]. Furthermore, Flnc-knockdown myoblast cells exhibited decreased fusion and rounded fibers, revealing the essential role of Flnc in muscle myogenesis and viability in mice [197]. In addition, Flnc has been reported to associate with muscle-specific protein HSPB7 or disheveled-2 (Dvl2), which activates the Wnt/β-catenin signaling pathway, crucial for the regulation of skeletal muscle development [198,199]. Flnc deficiency has been linked to muscle atrophy, while its overexpression has been observed to cause muscle hypertrophy in both in vivo and in vitro models [198,199]. Furthermore, the lack of interaction with KY protein due to Flnc hyperactivation in kyphoscoliosis (KY)-deficient mice has implications in muscular dystrophy [200]. On the other hand, Ruparelia et al. have identified that protein dysfunction and impaired autophagy could manifest as Flnc myofibrillar myopathy [201]. This indicates that modulations in Flnc-mediated autophagy could potentially affect skeletal muscle development, offering a new avenue for exploring therapeutic interventions. 

#### 3.6.2. Fascin 

Fascin is a crucial actin-bundling protein that enhances and stabilizes F-actin filaments and actin-based cellular protrusions [202]. This protein is pivotal in maintaining cellular structure, and its significance is underscored by its role in cellular metastasis-associated invasion [203,204]. Fascin can be categorized into three groups based on their amino acid sequences. Among these, fascin-1, a ubiquitous type, has been observed to slide as fast as single actin filaments on myosin II and myosin V through interactions with other ABPs in human skeletal muscle [205]. This characteristic indicates fascin’s integral role in modulating actin dynamics within skeletal muscle cells. Moreover, fascin is essential for the fusion of myoblasts in C2C12 cells and plays a pivotal role in muscle development in *Drosophila* [129,206,207,208,209]. Mechanically, it regulates myoblast fusion and myotendinous junction (MTJ) structure, acting as a crucial mediator in muscle cell interactions and developmental processes. The disruption of MTJ integrity due to prolonged depletion of fascin has profound implications, having a more significant effect on animal survival than the transient depletion that disrupts fusion [207]. Unveiling the detailed mechanisms and interactions of fascin could provide insights into developing therapeutic strategies for conditions associated with cellular structural disruptions and skeletal muscle disorders.

### 3.7. F-Actin Stabilizing Proteins

F-actin-stabilizing proteins can bind to the sides of actin filaments and regulate polymerization and depolymerization. Interestingly, some of them have a critical function in regulating skeletal muscle development, for example, Calponin (CNN), Drebrin (Dbn), XIN-repeating protein (XIN), and Microtubule Associated Monooxygenasep 2 (MICAL2).

#### 3.7.1. Calponin (CNN)

CNN, identified initially as an F-actin binding protein, is characterized by three isoforms constituting the CNN family, each marked by the N-terminal Calponin [61]. The isoform, notably CNN3, regulates trophoblastic cell fusion and actin cytoskeleton rearrangement, especially during actin stress fiber remodeling [61]. Mutations in CNN3 have been linked to neonatal lethality and extensive malformations in mice embryos due to induced actin cytoskeletal reorganization [210]. In addition, CNN3 has been observed to be upregulated during muscle development and regeneration in various species, including pigs and mice, hinting at a potential association with muscle development [211,212]. Interestingly, silencing CNN3 using siRNA dramatically repressed the expression of proliferation-related genes, such as cyclin D, CDK2, CDK4/6, Ki67, and MyoD [211,212]. Besides, the knockdown of CNN3 dramatically suppresses the expression of differentiation marker proteins (MEF2A, MyoG) and myotube formation [211,212]. Studies by She et al. revealed that the inhibition of CNN3 protein synthesis is associated with a negative correlation with AMPK/mTOR and Akt/mTOR signaling pathways impacting the proliferation and differentiation of C2C12 myoblasts [211]. In contrast, other studies found that ROCK activation at the onset of differentiation can phosphorylate CNN3 at Ser293 (pSer293) and Ser296 (pSer296), which triggers cytoskeletal rearrangement necessary for myotube formation [213,214]. Moreover, suppression of CNN3 by knockdown of ROCK1/2 accelerates myogenin and skeletal myosin expression, whereas the CNN3 mutant lacking Ser293/296 or ROCK knockdown suppresses skeletal myogenesis [214]. The contrasting findings necessitate further in-depth studies to reconcile the varying impacts of CNN3 modulation in different cellular contexts and to elucidate the comprehensive roles and mechanisms of Calponin in skeletal muscle development and other cellular processes.

#### 3.7.2. Drebrin (Dbn1)

Dbn1, an F-actin-binding protein, is pivotal in myogenic differentiation, acting as a promoter of this process [62]. Its function and regulation, particularly in relation to various signaling pathways, are integral to our understanding of muscle development and the complexities of cellular differentiation. Dbn1 expression has been observed to increase during myogenic differentiation, suggesting its role as a promoter of the differentiation process [215]. This promotion occurs in a p38 MAPK-dependent manner [215]. Treatment with SB203580, the p38 inhibitor, mediated Dbn1 reduction, leading to suppression of myoblast differentiation by inhibiting MyoD and MyoG expression and myotube formation in C2C12 myoblasts [215]. Thus, it is proposed that inhibition of Dbn1 might suppress myoblast differentiation, potentially by inducing cell proliferation or apoptosis [215]. Several pathways and interacting proteins have been proposed to explain the phenotype associated with Dbn1. Homer, for instance, is known to regulate myoblast differentiation and fusion [216,217]. It acts as a scaffold protein, enabling cdc42 to interact with Dbn1, regulate actin polymerization, and participate in filopodia formation [218]. Furthermore, the microtubule plus-tip protein EB3 is known to coordinate F-actin–microtubule interactions, controlling neuritogenesis by binding to Dbn1 [219]. EB3 has also been reported as a transcriptional regulator during myogenic differentiation, affecting microtubule organization [220]. Deletion of EB3 has been associated with microtubule disorganization in the myoblast stage and a significant reduction in myoblast fusion [220]. The signaling interactions with Dbn1 suggest a potential regulatory mechanism of Dbn1 in myogenesis. The interactions and regulatory mechanisms involving Dbn1, EB3, and other proteins illustrate the complex network that governs myogenic differentiation and myoblast fusion.

#### 3.7.3. Xin Actin-Binding Repeat-Containing Protein 1 (Xin)

Xin actin-binding repeat-containing protein 1 (Xin), also known as cardiomyopathy-associated-1 (Cmya-1), has been recognized for its ability to bind F-actin and protect against depolymerization [66]. Accumulated studies have documented that Xin is a valuable biomarker of muscle damage and myopathy [221]. Significant upregulation of Xin is induced during the initial phases of muscle satellite cell regeneration, and its expression is lower in adult skeletal muscles [222,223]. The overexpression of Xin has been associated with the promotion of myogenic-related gene expression, including MyoD, Myf5, and MEF2. Conversely, the reduction of endogenous Xin is linked to the facilitation of cell proliferation and migration and an increase in the MyHC turnover rate in C2C12 myoblasts [224]. Several hypotheses have emerged regarding the regulatory mechanism of Xin in muscle damage and wasting, suggesting interactions with other partners such as Ena/VASP homology 1 and filamin C. Xin, characterized by a highly conserved structure with a proline-rich domain, can directly interact with Ena/VASP homology 1 via binding to proteins containing consensus (EVH1) domains and with Flnc via the C-terminus [225]. A study showed that Flnc is slightly increased during skeletal muscle regeneration in injury [226] and in activated myogenic progenitor cells and newly regenerated myotubes, aligning with the expression patterns of Xin in dystrophic ky/ky muscles and skeletal muscle regeneration [224]. Thus, the conserved structure and diverse interactions of Xin offer valuable insights into the understanding of muscle regeneration mechanisms and the development of potential therapeutic strategies for muscle damage and myopathies.

#### 3.7.4. Nexilin (NEXN)

NEXN is a F-actin-binding protein ubiquitously found in the sarcomeric Z-disc of cardiac and skeletal muscle tissues, a crucial structural interface between the sarcolemma and the cytoskeleton [65,227,228]. Its dynamic expression and interactions in muscle tissues under varying physiological conditions underscore its importance in muscle development and potentially in muscle atrophy. In response to bed rest-induced atrophy, particularly in aging, NEXN has been observed to be downregulated [229], contrasting its upregulation in muscles following 24 h of exercise [230], indicating that NEXN may have a prominent role in muscle development and adaptation to different physiological conditions. NEXN expression is increased in fast-growth chicken muscles compared to slow-growth ones, further emphasizing its role in muscle development and growth [199]. Studies by Lee et al. have elucidated that, in the basal state of skeletal muscle L6 myotubes, NEXN is stably associated with IRS1 [231]. The disassembly of this complex, either by insulin or by silencing NEXN, stimulates the IRS1/PI3K/Akt signaling pathway and facilitates glucose uptake in L6 cells [231]. Additionally, the exposure of L6 myotubes to latrunculin disrupted the spatial patterning of NEXN and blocked the dissociation with the IRS1/NEXN complex in the presence of insulin, shedding light on the regulatory role of actin dynamic reorganization in this complex’s efficient dissociation [231]. Gardner et al. have reported the pivotal function of p38α/β in muscle cell fusion and highlighted that the inhibition of p38α/β could reduce skeletal muscle differentiation in vitro [65]. Notably, the treatment with SB202190 in combination with IGF-1 demonstrated rescues in myotube formation, and the removal of SB202190 induced NEXN upregulation and increased myotube fusion [232]. These observations suggest that NEXN plays a vital role in muscle development. Further studies are imperative to delve deeper into the regulatory mechanisms of NEXN in muscle atrophy and to explore its potential implications in muscle-related disorders.

#### 3.7.5. [F-Actin]-Monooxygenase MICAL2

MICAL2 is critical in regulating actin dynamics by inducing redox reactions in F-actin, which modulates actin filament polymerization and turnover [63,64]. MICAL2 is ubiquitously expressed in the skeletal muscles of *Drosophila* [233,234] and mice [235]. It has been notably upregulated in a mouse Duchenne muscular dystrophy (DMD) model, emphasizing its potential role in muscle disorders [236]. Studies by Giarratana et al. reported significant increases in MICAL2 expression during skeletal muscle differentiation and regeneration [235]. It is predominantly localized in the nucleus during the fusion stage in C2C12 and satellite cells [235]. The silencing of MICAL2 inhibited skeletal muscle differentiation and induced degeneration in cells, reinforcing its role in muscle development [235]. The translocation of MICAL2 from the cytoplasm to the nucleus may offer redox alteration of nuclear actin and associated activation of myogenic transcription factors [235]. Thus, this translocation and its subsequent effects indicate the therapeutic potential of MICAL2 for muscle disorders. The modulation of MICAL2 can potentially influence muscle differentiation and regeneration, offering insights into developing targeted approaches for muscle-related disorders.

### 3.8. Muscle Contractile-Related ABPs in Myogenic Differentiation

#### 3.8.1. α-Actinin

α-Actinin, along with myosin IIs (NM IIs), nebulin, and tropomyosin, is a specific protein expressed in mature skeletal muscle tissue and is instrumental in muscle contraction when coupled with myosin motors [67,68,69,70,237]. These proteins, pivotal in the architecture and function of mature skeletal muscles, are also implicated in Nemaline myopathies (NM) and are under investigation for potential clinical therapies [237]. Their expression and function during myogenic differentiation emphasize their relevance as mature muscle genes.

α-Actinin serves as an essential structural protein, organizing and stabilizing the sarcomere, the fundamental contractile unit of muscle cells [238]. It plays a vital role in linking actin filaments at the Z-discs, anchoring the thin filaments, and providing structural support to the sarcomere [238]. The expression of α-actinin is initiated early, in day-one cells, and progressively increases throughout the differentiation process [238], emphasizing its continual involvement in muscle development and maturation. Swailes et al. showed the role of NM IIs in promoting the alignment and fusion of myoblasts to form multinucleated myotubes [239]. NM II-A and -II-B, being localized in the Z-line and intercalated disc of human skeletal muscle, hint at their intricate involvement in the contraction/relaxation mechanism of muscles [239]. During myofiber development, newly formed myoblasts fuse with the growing ends of the myotube, culminating in a mature and fully differentiated myofiber where mature isoforms of skeletal muscle myosin supersede the preceding NM II [240]. Given the involvement of α-Actinin and associated proteins in Nemaline myopathies, ongoing research is imperative to elucidate potential clinical therapies targeting these proteins. Their pivotal roles in muscle contraction and structural integrity make them promising targets for therapeutic interventions to mitigate muscle-related disorders.

#### 3.8.2. Nebulin

Nebulin, a significant protein in skeletal muscle, is renowned for its large molecular weight ranging from 700–800 kDa. Constituting approximately 2–3% of the myofibrillar protein mass, it has intricate interactions with other muscle proteins, making it crucial for muscle structure and function. The architecture of Nebulin is arranged to precisely interact with individual monomers of the actin filament, ensuring a coordinated association with a single tropomyosin (Tm)/troponin (Tn) complex. The N-terminal modules of nebulin feature a binding site dedicated to tropomodulin, a capping protein that binds to the pointed end of the thin filament. This unique organization underscores nebulin’s role as a scaffold and regulator of skeletal muscle’s thin filaments. Research has demonstrated the role of nebulin in regulating the length of thin filaments in vivo [70,241]. Specifically, skeletal muscles devoid of nebulin display shorter, thin filaments, highlighting its significance in maintaining the structural integrity and functional efficiency of muscle fibers [70,241]. Furthermore, recent studies have delved into the indirect influences of proteins on sarcomere assembly. Immunohistochemical analysis and quantitative PCR have revealed that PFN2a detrimentally affects sarcomere assembly by indirectly suppressing the mRNA levels of several skeletal muscle proteins, including α-actinin, titin, nebulin, and tropomyosin 1. This suppression, in turn, hinders C2C12 myogenic differentiation, emphasizing the intricate regulatory networks governing muscle development and differentiation [83]. Understanding the complex roles and interactions of nebulin can provide invaluable insights into muscle development, function, and related disorders (Table 2).

## 4. Epigenetic Regulation of ABPs by ncRNAs in Skeletal Myogenesis

**Figure 3:**
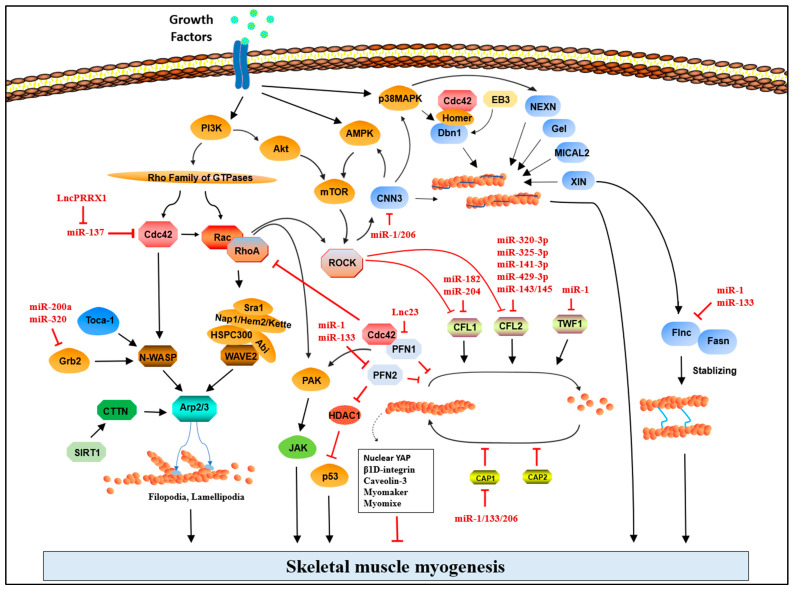
Roles of ABPs and their regulation by non-coding RNAs in skeletal myogenesis.

The expression of proteins is differentially regulated by epigenetic factors in normal and pathological conditions. There has been significant progress in understanding the association between ncRNAs, such as lncRNAs and miRNAs, and a wide range of human diseases in the past decade [244,245,246,247,248]. In particular, the regulatory circuitry of ncRNAs in skeletal myogenesis has been extensively studied in conjunction with essential genes controlling muscle homeostasis [249]. In this point of view, a number of ncRNAs were found to regulate ABPs at transcriptional and post-transcriptional levels during skeletal myogenesis (Figure 3). This section summarizes (Table 3) and discusses the critical ncRNAs that target ABP and their involvement in skeletal myogenesis.

Lnc23 is a specific lncRNA, predominantly localized in the nucleus, which has emerged as a significant regulator of myogenesis in bovine myoblasts [250]. Studies reveal that Lnc23 is a pivotal player in skeletal muscle growth and development. Its importance is particularly highlighted in the regulation of PFN1 [250], a protein known to modulate myogenic differentiation by repressing RhoA and Rac1 at the transcriptional level [79,80]. Lnc23 has been demonstrated to suppress PFN1 expression directly, subsequently weakening the inhibitory effect of PFN1 on RhoA and Rac1 [250]. This modulation promotes myogenic differentiation through the SRF and β-catenin/TGF signaling pathways [250]. Additionally, lnc23 fosters the expression of Rac1, a member of the Rho family of GTPases involved in actin polymerization. This promotion occurs through the actin polymerization-mediated Activation of the Arp2/3 complex, which, in turn, accelerates myogenic differentiation [250].

MiR-1/-133/-206 are miRNAs highly expressed in muscle and modulate the expression of target genes associated with proliferation and differentiation in skeletal muscles [212,251,252]. Mishima et al. revealed that miR-1 and miR-133 regulate actin organization during sarcomere assembly by targeting actin-related genes, including CNN3, PFN2, and the Arp2/3 complex [251]. In addition, miR-1 and miR-206 were found to repress CNN3 expression at transcriptional and translational levels, thus negatively regulating skeletal myogenesis [212,253]. A pivotal study highlighted that the post-transcriptional downregulation of endogenous CAP1 expression by canonical miR-1/-133/-206 led to an increase in F-actin accumulation and, subsequently, a reduction in myogenic differentiation in both human LHCN-M2 and murine C2C12 myoblasts [100]. 

Throughout the differentiation process, a decrease in MRTF-A protein levels was observed, pointing towards the significant role of post-transcriptional regulation of MRTF-A by translational repression during myogenic differentiation [117,118]. Previous research has identified a variety of myo-miRs, including miR-1a/b, miR-133, miR-206, miR-208b, miR-486, and miR-499, each of which has multiple targets [254]. Subsequent studies have confirmed the role of previously identified myo-miRs and expanded the potential myo-miRNome to encompass 33 miRNAs markedly upregulated upon the differentiation of C2C12 cells [255]. On the other hand, among the miRNAs known to regulate MRTF-A, namely, miR-1a-3p, miR-206-3p, and miR-219a-5p [256,257], only miR-1a-3p and miR-206-3p were significantly enriched with the *MRTF-A* 3′UTR and selectively enhanced upon differentiation [255]. Interestingly, miR-486 was transcriptionally upregulated by MRTF-A/SRF [192], suggesting a regulatory feedback loop involving MRTF-A/SRF and miR-486. 

As previously described, the reduction of nonmuscle CFL1 levels can facilitate skeletal muscle myogenic differentiation [89]. MiR-182 can target *CFL1* 3′UTR and suppress CFL1 expression, thereby positively regulating skeletal muscle growth and development [89]. Sevoflurane-induced miR-204, another regulator of myogenic differentiation [188,189], has been shown to improve the function related to myocardial ischemia/reperfusion injury in mice by targeting and suppressing CFL1 [258]. In contrast to CFL1, CFL2 is a critical marker for skeletal muscle and has drawn significant interest in recent years. Nguyen et al. have demonstrated that several miRNAs, such as miR-141-3p, miR-320-3p, miR-325-3p, and miR-429-3p, can suppress CFL2 expression and promote abnormal F-actin formation [259,260,261,262]. This process facilitates the translocation of YAP1 from the cytoplasm to the nucleus, inhibiting myogenic differentiation [259,260,261,262]. The upregulation of miR-141-3p has been observed in numerous muscle-wasting conditions, such as mitochondrial dysfunction, oxidative stress, and endoplasmic reticulum stress [263]. Conversely, Chang et al. have demonstrated the opposite role of miR-320 in C2C12 myoblast differentiation by directly targeting Grb2 [264]. Overexpression of miR-320 reduced endogenous Grb2, subsequently promoting myoblast differentiation [264]. Another study has reported that Grb2 was upregulated, whereas miR-200a was downregulated, in chicken breast muscle, indicating that miR-200a might be involved in cell differentiation and proliferation via the direct regulation of Grb2 expression [265]. Recent studies have indicated that miR-143 and miR-145 play roles in multiple cellular signaling pathways to regulate skeletal muscle cell proliferation and differentiation [266,267,268,269]. This suggests a potential interference of miR-143/145 on CFL2 expression in the regulation of skeletal myogenesis. 

Another candidate for targeting actin-related genes, miR-137, was reported to inhibit proliferation markers, including PCNA, CCNB1, and CCND2, at both mRNA and protein levels by directly targeting Cdc42 3′UTR [270]. Additionally, lncPRRX1 is involved in bovine skeletal muscle development and promotes the proliferation of bovine myoblasts. The study found that lncPRRX1 could act as a competitive endogenous RNA (ceRNA) sponge against miR-137, thereby promoting Cdc42 activation and proliferation of bovine myoblasts [270]. 

The intricate interplay between these miRNAs and their target genes, coupled with the involvement of feedback mechanisms, underscores the finely tuned regulation of myogenic differentiation. It also highlights the promising utility of these miRNAs in potential therapeutic approaches for muscle-related disorders. To gain a deeper comprehension of their implications in muscle physiology and pathology, it is imperative to conduct further comprehensive studies. These studies will help unveil the broader significance and prospective applications of these miRNAs, ultimately enriching our understanding of muscle development and diseases. Furthermore, deciphering the regulatory mechanisms that govern these miRNAs offers valuable insights into muscle biology and holds promise for developing diverse therapeutics for muscle diseases.

**Table 3:** Summary of ncRNAs targeting ABPs in skeletal myogenesis.

lncRNAs/miRNAs	Functions	Mechanisms	Refs.
lnc23	Promotes myogenic differentiation	Lnc23 may release RhoA and Rac1 from the inhibitory effect of PFN1 by binding to PFN1	[250]
miR-1/miR-133	Induces actin disorganization during sarcomere assembly	Target PFN2, Arp2/3	[251]
miR-1	Negatively regulates skeletal muscle development	Targets CNN3	[212]
miR-206	Glycolysis regulator during prenatal skeletal muscle development		[212,253]
miR-182	Promotes bovine primary myoblast differentiation	Targets CFL1	[89]
miR-204	Improved in myocardial ischemia/reperfusion injury in mice		[258]
miR-320-3p	Negatively regulates myogenic differentiation	Targets CFL2	[259]
miR-141-3p	Negatively regulates myogenic differentiation		[262]
miR-325-3p	Negatively regulates myogenic differentiation		[261]
miR-429-3p	Negatively regulates myogenic differentiation		[260]
miR-145	Increases stress fiber formation by modulating actin dynamics and cytoskeletal assembly		[271]
miR-1/133/206	Promotes myogenic differentiation	Targets CAP1	[100]
miR-1/206miR-486	Promotes myogenic differentiation	MRTF-A	[192]
lncPRRX1/ miR-137	Promotes myogenic differentiation	lncPRRX1 repaired the defects in Cdc42 protein levels caused by miR-137	[270]
miR-320	Promotes myoblasts differentiation	Targets Grb2	[264]
miR-200a	Promotes cell differentiation and proliferation		[265]

## 5. Conclusions and Perspectives

Understanding the intricacies of myogenesis and its homeostasis holds significant potential for advancing therapeutic options and treatment strategies for skeletal muscle disorders. It has long been believed that investigating and modulating signaling molecules affecting the quantity and quality of protein synthesis has been the cornerstone of addressing muscle atrophy. Despite this, the effective and safe treatment of skeletal muscle disorders remains challenging and unsolved. In the past decade, researchers have diligently studied the molecule that links actin cytoskeleton dynamics with myogenic programs. Due to this, the exploration of specific mechanisms controlling myogenic processes mediated by ABPs has recently emerged as a promising avenue of research. 

This comprehensive review discussed critical ABPs for actin remodeling, which profoundly impact cell proliferation, differentiation, and fusion into muscle fibers. Dysfunction of these ABPs disrupts signaling pathways necessary for muscle development, leading to muscle disorders. Furthermore, we have reviewed critical non-coding RNAs (ncRNAs), elucidating their potential to modulate ABPs and specific signaling pathways. Recent advancements in ncRNA research and deep sequencing techniques promise to significantly expand our understanding of the roles and significance of ncRNAs in the future. To achieve this objective, it is imperative to gain a deeper understanding of the intracellular signaling pathways governed by the ABP that govern myogenic processes. Although recent studies demonstrate a direct relationship between ABPs and myogenesis, there are still a number of questions that need to be answered. Firstly, it is unclear how ABPs regulate myogenesis in detail and in a concrete way. Secondly, many ABPs have only been studied in vitro, which raises questions about their consistency with in vivo or clinical observations. Additional research is necessary to determine whether the dysregulation of ABPs is causally linked to muscle disorders in animal models and clinical stages. Finally, it is still unclear which mechanisms control ncRNA expression, even though they target ABPs. This deeper comprehension will facilitate the development of strategies for addressing a broad spectrum of muscular disorders.

## Figures and Tables

**Table 2:** Expression of ABPs and their roles in skeletal myogenesis.

Protein Groups	ABPs	Expression	Function	Mechanism	Refs.
Proteins regulating F-actin assembly	PFN1	Expressed early in muscle development	Negatively regulates myogenic differentiation.	Represses RhoA and Rac1 at transcriptional levels	[76,77]
				Binds to Cdc42 activity, activates PAK/JNK signaling pathways	[81]
	PFN2	Expressed early in development	Inhibits cell proliferation while inducing apoptosis, represses the myogenic differentiation of C2C12 myoblasts	Reduces HDAC1 localization and subsequently induces p53 Expression	[83]
Proteins regulating F-actin disassembly	CFL1	Expressed in the early stage of differentiation and declined during muscle development	Overexpression of CFL1 suppresses the differentiation of bovine primary myoblasts.	Increases actin depolymerization	[89]
	CFL2	Expressed later and increases during muscle differentiation and fusion	CFL2-knockout mice lead to an abnormal accumulation of F-actin and progressive disturbance of the sarcomeric architecture of skeletal muscles.	Decreases p38 MAPK, CBP, AMPKα1, and MEF2C pathway and increases ERK2 expression	[93,94,95,96]
			CFL2 knockdown in C2C12 myoblast cells inhibits myogenic differentiation and promotes cell proliferation.	Abnormal F-actin formation modulates nuclear YAP localization	[26,27]
	WDR1	Highly expressed during exercise after muscle disuse	WDR1-knockout mice cause postnatal lethality, sarcomere disorganization, and contractility defects.	Actin aggregate formation	[81,97]
	CAP1	Downregulated during myogenic differentiation.	CAP1 deletion results in a spread-out morphology, increased cell size and nuclei, and inversely correlating with myogenic differentiation.	Regulates F-actin organization Regulate the expression of myoblast profusion molecules, such as β1D-integrin, Caveolin-3, Myomaker, and Myomixe.	[100]
	CAP2	Increased upon differentiation in vitro or regeneration in vivo	Knockout CAP1 in mice is characterized by delayed maturation of motor functions, reduced muscle strength, and weakness.	α-SMA and α-CAA, an internal piece of sarcomere F-actin accumulates, thereby leading to ring fibers	[28]
	TWF1	Increased in the early stage of myoblast fusion, then gradually decreased	Its knockdown promotes cell proliferation and inhibits myogenic differentiation.	TWF1 knockdown accumulates F-actin, leading to nuclear YAP localization	[81]
Monomer-sequestering proteins	Tb4	Upregulated during myotube differentiation of C2C12 cells	Promotes new myofiber formation	Intracellular G-actin-sequestering and inhibits the assembly of actin fibers	[111,112]
	MRTF-A	Expressed in the early stage of differentiation and declined during differentiation	MRTF-A promotes differentiation of myoblasts and the expression of MyoD and MyoG	Rho/MRTF/SRF pathway and regulate Pax7 expression	[117,118,127]
Proteins for nucleation sites in actin branching	Arp2/3	Expressed in skeletal muscles	Mutants in Arp2/3 have a fusion block and foci phenotype	Inhibits actin polymerization in myoblast fusion	[129]
	N-WASP	Expressed in the early phase of mouse embryonic development	Knockout of N-WASP causes early embryonic lethality, characterized by developmental delay	Inhibits the formation of filopodia and lamellipodia, which are required for cell movement.	[141]
			Knockout N-WASP in myoblasts fail to fuse and form multinucleated myotubes		[136,137]
	WAVE2	Concentrated in the leading edges of lamellipodia in myoblasts	Promotes myoblast fusion, promotes lamellipodial formation and subsequent migration in C2C12 cells	Acts downstream of HGF/PI3K	[29,30]
	Fhod3	Accumulated in skeletal muscles	Flight muscle myofibrils were disrupted in muscle-specific silencing of Fhos in *Drosophila*.	Sarcomere organization	[151,152]
	DAAM1	Accumulated in skeletal muscles	Absence of DAAM1 results in disorganization of thin filaments, leading to shorter and sparsely distributed sarcomeres.	Regulated F-actin content	[29,31]
	Dia	Accumulated in skeletal muscles	Regulating the length and width of each sarcomere during flight muscle development, required during myoblast fusion	Regulated F-actin content	[136,154]
	CTTN	Expressed in skeletal muscles	Facilitates GLUT4myc translocation in L6 myotubes and C2C12 cells	F-actin and stress fiber acculation	[168,169]
Actin nucleation activators	Cdc42/Rac	Expressed in skeletal muscles	Cdc42/Rac mutated embryonic mice exhibited thinner muscle fibers and myoblast fusion.	Regulates the expression of F-actin/Arp2/3 complex	[142]
	Grb2	Expressed in skeletal muscles	Grb2 mediates the inhibition of myogenic differentiation by enhancing actin polymerization.	Regulates N-WASP by direct binding	[137]
	Toca-1	Expressed in skeletal muscles	Reduction of Toca-1 in C2C12 cells leads to a substantial decrease in myogenic differentiation.	Regulates N-WASP	[148]
Capping proteins	Gel	Induced steadily during myoblast differentiation	Limits myoblast fusion in *Drosophila*	Binding to F-actin in the barbed end	[175]
	CapZ	Upregulated in myotubes	Overexpressed CapZ-β2 results in severe myofibril disruption and, ultimately, death	Sarcomere organization	[177]
	Tmod1	Decreased during differentiation	Significantly inhibits myogenic differentiation	Reducing the expression of muscle-specific genes	[136,183,185]
	Tmod4	Decreased during differentiation	Significantly inhibits myogenic differentiation	MEF2A can bind to the promoter region of Tmod4 and inhibit Tmod4 expression	[182]
	Lmod3	Induced and promotes myoblast differentiation	Loss of Lmod3 function leads to lethal nemaline myopathy.	Sarcomere organization and regulated by MRTF/SRF and MEF2	[190]
Actin filament cross-linking proteins	Flnc	Expressed early and decreases during cell differentiation	Regulates skeletal muscle development. Flnc-knockdown cells decreases myoblast fusion and rounded fibers.	Associated with the muscle-specific protein HSPB7 or disheveled-2 (Dvl2), which induces the wnt/β-catenin signaling pathway	[29,197,198,199]
	Fascin	Increased during differentiation	Depletion of fascin disrupts MTJ integrity	Slide as fast as single actin filaments on myosin II and myosin V	[129,205,207,208,209]
F-actin stabilizing proteins	CNN3	Increased during muscle development and regeneration in pigs and mice	Knockdown CNN3 dramatically suppresses differentiation.	Akt/mTOR and AMPK/mTOR, ROCK	[211,214]
	Dbn1	Increases during myogenic differentiation	Inhibition of Dbn1-induced cell proliferation or apoptosis, suppresses myoblast differentiation.	p38 MAPK-dependent manner, Homer/Cdc42, EB3	[215,216,217,218,220]
	Xin	Upregulated during the early phases of skeletal muscle regeneration	Reduced proliferation and apoptosis, and promotes myogenic differentiation	Works with other partners, such as Ena/VASP homology 1 and Flnc	[224]
	NEXN	Highly expressed in the heart and skeletal muscles. Increased in fast-growth compared to slow-growth chicken muscle	Promotes glucose uptake, and myotube formation in L6 cells	IRS1/NEXN complex negatively regulates the IRS1/PI3K/Akt signaling pathway	[229]
			Promotes myotube fusion C2 myoblasts	Association IGF-I/p38α/β/kinase-mediated signaling	[232]
	MICAL2	A significant increase in myogenic differentiation and skeletal muscle regeneration	Promotes degeneration and regeneration	Redox alteration of nuclear actin and associated activation of myogenic transcription factors	[236]
Muscle contractile-related ABPs in myogenic differentiation	α-Actinin	Gradually increased during the differentiation	Links actin filaments at the Z-discs, imparting structural reinforcement and serving as an anchor for the thin filaments	Organizing and stabilizing the sarcomere	[238]
	NM II-A and -II-B	Expressed in fully differentiated myofiber	Assists the fusion of newly generated myoblasts		[239,240]
	Nebulin	Expressed in skeletal muscle	Interact with individual monomers of the actin filaments		[70,241]
	Tpms	Accumulated in skeletal muscles	Regulate muscle remodeling/regeneration		[242,243]

## Data Availability

Not applicable.

## Abbreviations

ABPs	Actin-binding proteins
Abi	Abl interactor 1 also known as Abelson interactor 1
Abp1	*S. cerevisiae* actin-binding protein-1
ADF	Actin depolymerization factor
Akt	Serine/threonine kinase
AMPK	AMP-activated protein kinase
Arp2/3	Actin-related protein-2/3
BMP	Bone Morphogenetic Proteins
CAP	Cyclase-associated protein
CapZ	Capping protein
Cdc42	Cell Division Cycle 42
CFL	Cofilin
CNN	Calponin
CTTN	Cortactin
Dbn	Drebrin
Des	Destrin
Dvl2	Disheveled-2
EB3	End-binding 1
EGFR	Epidermal growth factor receptor
EVH1	Ena/VASP homology 1
Fln	Filamin
Fscn	Fascin
Gel	Gelsolin
Grb2	Growth factor receptor-bound protein 2
HDAC1	Histone Deacetylase 1
HSPB7	Heat Shock Protein Family B member 7
HSPC300	Haematopoietic stem/progenitor cell protein 300
IGF-1	Insulin-like growth factor 1
IRS1	Insulin receptor substrate 1
IRSp53	Insulin receptor substrate protein 53kDa
JAK/STAR	Janus kinase/signal transducer and activator of transcription
LncRNAs	Long non-coding RNAs
Lmod	Leiomodin
MCK	Muscle creatine kinase
MHC	Myosin heavy chain
MICAL	Microtubule Associated Monooxygenasep
MicroRNAs	miRNAs
Mrf4	Myogenic regulatory factor 4
MRFs	Myogenic regulatory factors
mTOR	Mammalian target of rapamycin
Myf5	Myogenic factor 5
MyoD	Myogenic factor 3
MyoG	Myogenin
Nap1/Hem2/Kette	Nck-associated protein 1/Hematopoietic protein 2/Kette
Nck	Non-catalytic region of tyrosine kinase
ncRNAs	Non-coding RNAs
NEXN	Nexilin, Nelin
NF-κB	Nuclear factor kappa-light-chain-enhancer of activated B cells
N-WASP	Neural- WASP
p38 MAPK	p38 mitogen-activated protein kinases
PAK/JNK	p21-activated kinase/c-Jun amino terminal kinase
Pax7	Paired box 7
PFN	Profilin
PI3K	Phosphatidylinositol 3-kinase
RhoA/Rac1	Ras homolog family member A/Rac Family Small GTPase 1
ROCK	Rho-associated protein kinase
SCAR	Suppressor of cyclic AMP repressor
Shh	Sonic Hedgehog
SIRT1	Sirtuin 1
Sra1/Cyfip1	Steroid receptor RNA activator 1/Cytoplasmic FMRP Interacting Protein 1
SRF	Serum Response Factor
TGFβ	Transforming growth factor β
Tmod	Tropomodulin
Tβ4	Thymosin β4
Toca	Transducer of Cdc42-mediated actin assembly
Tpms	Tropomyosins
TWF	Twinfilin
WASP	Wiskott—Aldrich syndrome protein
WAVE	WASP family verprolin homologous protein
WDR1	WD-repeat domain 1
WIP	WASP-interacting protein
WRC	WAVE regulatory complex
XIN	XIN-repeating protein
YAP	Yes-associated protein 1
α-CAA	α-smooth muscles actin
α-SKA	α-skeletal muscle actin
